# From menses to ovulation: the immunomodulatory role of monocyte cytokines in menstrual cycle physiology and reproductive health – a narrative review

**DOI:** 10.1097/MS9.0000000000004021

**Published:** 2025-10-07

**Authors:** Emmanuel Ifeanyi Obeagu, Getrude Uzoma Obeagu

**Affiliations:** a Department of Biomedical and Laboratory Science, Africa University, Mutare, Zimbabwe; b School of Nursing Science, Kampala International University, Kampala, Uganda

**Keywords:** cytokines, immune regulation, menstrual cycle, monocytes, reproductive health

## Abstract

Monocytes, as essential components of the immune system, play a pivotal role in regulating immune responses throughout the menstrual cycle. Their cytokine secretion is integral to immune modulation, influencing processes like endometrial remodeling, ovulation, and implantation. Cytokines such as interleukins, tumor necrosis factor (TNF), and growth factors secreted by monocytes help maintain a delicate balance between inflammation and immune tolerance, crucial for reproductive health. This balance facilitates both tissue repair and immune protection, ensuring proper function of the reproductive organs during the menstrual cycle. Dysregulation in monocyte cytokine secretion can contribute to a range of reproductive disorders, including endometriosis, polycystic ovary syndrome, and recurrent pregnancy loss. An overproduction of pro-inflammatory cytokines, such as TNF-α and interleukin-6, may lead to chronic inflammation, tissue damage, and impaired fertility. Conversely, inadequate secretion of anti-inflammatory cytokines can hinder the immune tolerance necessary for embryo implantation, potentially leading to complications like miscarriage. This review explores the pivotal role of monocytes and their cytokine secretion in maintaining reproductive health and their contribution to reproductive disorders.

## Introduction

Monocytes are a crucial subset of white blood cells that play a central role in the immune response and homeostasis. They are produced in the bone marrow and circulate in the blood before migrating to tissues, where they differentiate into macrophages or dendritic cells, contributing to both innate and adaptive immunity. Monocytes secrete a range of cytokines that regulate inflammation, immune cell recruitment, and tissue repair. In the context of reproductive health, these cytokines have profound implications for the regulation of the menstrual cycle, immune tolerance during pregnancy, and the maintenance of a healthy immune environment in the reproductive organs. Monocytes’ ability to secrete cytokines and modulate immune responses makes them essential for the proper functioning of the reproductive system^[1–4]^. The menstrual cycle, a complex process orchestrated by hormonal changes, involves the dynamic interaction between the endocrine and immune systems. Monocytes are involved in the regulation of this process, as their cytokine secretion helps mediate various stages of the cycle, from endometrial shedding during menstruation to the potential for embryo implantation. Hormonal fluctuations, particularly the changes in estrogen and progesterone levels, influence the behavior of monocytes and their ability to secrete different cytokines in response to the immune needs of the reproductive tissues. For example, during the follicular phase, when estrogen levels rise, monocytes are more likely to secrete pro-inflammatory cytokines, facilitating tissue remodeling and preparation for implantation. In contrast, during the luteal phase, progesterone predominates, inducing a shift toward anti-inflammatory cytokines to promote immune tolerance and support pregnancy^[5–8]^.

**HIGHLIGHTS**

- Monocyte cytokines dynamically fluctuate across the menstrual cycle, influencing endometrial remodeling.
- Pro-inflammatory cytokines peak during menstruation to aid tissue shedding.
- Anti-inflammatory responses dominate peri-ovulatory phases for implantation readiness.
- Dysregulation in monocyte activity may impair fertility and menstrual health.
- Understanding cytokine patterns offers insights into reproductive immune balance and disorders.

Cytokines are small signaling molecules that mediate communication between immune cells and regulate a variety of physiological processes. The cytokines secreted by monocytes, including interleukins (ILs), tumor necrosis factor-alpha (TNF-α), interferons, and growth factors, have been shown to regulate inflammation, cell proliferation, angiogenesis, and tissue repair – all of which are essential functions during the menstrual cycle. Pro-inflammatory cytokines, such as IL-1β, IL-6, and TNF-α, help mediate the breakdown of the endometrial lining, enabling menstruation to occur. On the other hand, anti-inflammatory cytokines like IL-10 and transforming growth factor-beta (TGF-β) help promote tissue healing, suppress unnecessary immune activation, and maintain a tolerogenic immune environment in preparation for embryo implantation^[9,10]^. The interaction between monocytes and the reproductive system is not limited to immune regulation. Monocytes also play an important role in promoting fertility. For instance, their cytokine secretion influences the health and function of the ovaries, facilitating processes such as follicular development, ovulation, and luteal phase support. Abnormal cytokine secretion can disrupt these processes, leading to reproductive disorders such as anovulation, ovarian dysfunction, and infertility. Furthermore, the cytokines released by monocytes are involved in regulating the immune responses at the site of implantation, ensuring that the embryo is not rejected by the maternal immune system. This immune tolerance is crucial for the establishment and maintenance of a successful pregnancy^[11,12]^. Dysregulation in cytokine secretion by monocytes is implicated in a variety of reproductive health conditions^[13]^. For example, in endometriosis, an excess of pro-inflammatory cytokines such as TNF-α and IL-6 contributes to chronic inflammation and tissue damage, disrupting the normal function of the reproductive organs^[14]^. Similarly, in polycystic ovary syndrome (PCOS), there is often an imbalance in cytokine secretion that promotes a pro-inflammatory state, leading to insulin resistance, anovulation, and impaired fertility^[15]^. In recurrent pregnancy loss (RPL), improper immune responses due to dysregulated cytokine production by monocytes may hinder the process of embryo implantation or contribute to miscarriage^[16,17]^.

## Aim

The aim of this review is to explore the role of monocyte-derived cytokine secretion during the menstrual cycle and its implications for reproductive health.

## Monocyte cytokine secretion during the menstrual cycle

Monocytes play an essential role in immune regulation and tissue homeostasis, and their cytokine secretion during the menstrual cycle is a critical aspect of maintaining reproductive health. The menstrual cycle, regulated by hormonal fluctuations, requires a fine-tuned balance between immune activation and immune tolerance. Monocytes contribute significantly to this balance by secreting a wide array of cytokines, including pro-inflammatory cytokines (such as TNF-α, IL-1β, and IL-6) and anti-inflammatory cytokines (such as IL-10 and TGF-β), which regulate the processes of endometrial shedding, tissue repair, and immune tolerance during different phases of the cycle^[18,19]^.

## Follicular phase

During the follicular phase of the menstrual cycle, the body prepares for ovulation. Estrogen levels rise, and monocytes undergo functional changes that allow them to secrete pro-inflammatory cytokines, such as TNF-α, IL-1β, and IL-6. These cytokines play a critical role in initiating the breakdown of the endometrial lining, a process that is necessary for menstruation to occur in subsequent cycles. They also stimulate the recruitment of additional immune cells to the endometrium, contributing to the inflammation that is part of the remodeling process. Additionally, monocytes help in the preparation of the endometrial tissue for possible implantation by promoting angiogenesis and extracellular matrix remodeling through the secretion of growth factors like vascular endothelial growth factor^[20,21]^.

## Ovulation and luteal phase

Following ovulation, the luteal phase begins, marked by an increase in progesterone secretion from the corpus luteum. During this phase, the immune environment in the reproductive tract shifts toward an anti-inflammatory state. Progesterone exerts its influence on monocytes by modulating their cytokine profile, promoting the secretion of anti-inflammatory cytokines such as IL-10 and TGF-β. These cytokines help to suppress unnecessary immune responses and create a tolerogenic environment that is necessary for the embryo’s successful implantation and growth. This shift toward anti-inflammatory cytokines is vital to prevent the maternal immune system from attacking the embryo, which is genetically distinct from the mother^[22,23]^.

## Menstruation and endometrial repair

As menstruation begins, the immune system plays a crucial role in the shedding and repair of the endometrial lining. Monocytes secrete pro-inflammatory cytokines to help clear the tissue that is being sloughed off while also recruiting other immune cells, such as neutrophils and macrophages, to help with tissue remodeling and repair. In addition to inflammatory cytokines, growth factors secreted by monocytes facilitate the regeneration of the endometrial lining by promoting cell proliferation and migration. This balance of pro-inflammatory and anti-inflammatory responses is crucial to prevent excessive tissue damage and ensure proper healing of the uterine lining for the next cycle^[24,25]^.

## Dysregulation of cytokine secretion

In certain reproductive disorders, such as endometriosis and PCOS, cytokine secretion by monocytes becomes dysregulated. In endometriosis, for example, an excess of pro-inflammatory cytokines like TNF-α, IL-1β, and IL-6 leads to chronic inflammation and the formation of lesions outside the uterus, this can impair fertility. Similarly, in PCOS, an imbalance in cytokine production, with an overproduction of pro-inflammatory cytokines, contributes to insulin resistance, anovulation, and infertility. These conditions demonstrate the importance of maintaining the proper balance of cytokine secretion by monocytes to ensure optimal reproductive health^[26,27]^.

## Monocytes and immune regulation in reproductive health

Monocytes play a critical role in immune regulation, and their influence on reproductive health is both complex and vital for maintaining normal menstrual function, fertility, and successful pregnancy. As part of the innate immune system, monocytes are responsible for recognizing and responding to pathogen-associated signals and ensuring tissue homeostasis. They circulate in the blood and migrate to various tissues, where they differentiate into macrophages or dendritic cells, contributing to immune surveillance and regulation. In the context of reproductive health, monocytes help to modulate immune responses in the endometrium, ovaries, fallopian tubes, and placenta. This regulation is crucial for balancing immune activation and immune tolerance, which is essential for the proper function of the reproductive system^[28,29]^.

## Immune tolerance during pregnancy

One of the most important roles monocytes play in reproductive health is in promoting immune tolerance during pregnancy. The maternal immune system must differentiate between self and non-self to prevent the rejection of the embryo, which is genetically distinct from the mother. Monocytes contribute to this process by secreting anti-inflammatory cytokines, such as IL-10 and TGF-β, which suppress excessive immune activation and promote a tolerogenic environment in the uterus. These cytokines help modulate the maternal immune response, enabling the immune system to tolerate the presence of the embryo without mounting an immune attack. In addition, monocytes help to maintain a delicate balance between immune activation (necessary to defend against pathogens) and immune tolerance (necessary for embryo survival), ensuring a healthy pregnancy outcome^[12,30]^.

## Endometrial regulation and fertility

In the non-pregnant state, monocytes help regulate the endometrial cycle by participating in endometrial remodeling and immune cell recruitment. During the follicular phase of the menstrual cycle, monocytes secrete pro-inflammatory cytokines such as TNF-α and IL-1β, which are essential for the breakdown of the endometrial lining. This inflammatory response is critical for the shedding of the endometrium during menstruation and the preparation of the uterine tissue for the next cycle. However, this immune response must be carefully controlled to avoid excessive inflammation, which can lead to conditions like endometriosis or other reproductive disorders. As the cycle progresses into the luteal phase, monocytes shift their cytokine production toward anti-inflammatory cytokines such as IL-10, which helps maintain immune tolerance and support a favorable environment for embryo implantation^[31,32]^.

## Ovulation and ovarian function

Monocytes also influence ovarian function and the process of ovulation. They contribute to the regulation of ovarian steroidogenesis, follicular maturation, and the release of the oocyte during ovulation. Monocyte-derived cytokines can influence the expression of various enzymes involved in the synthesis of estrogen and progesterone, which are critical for preparing the uterus for implantation. Additionally, cytokines such as IL-6 and TNF-α play a role in the inflammatory processes that facilitate ovulation. An imbalance in the cytokine secretion by monocytes can disrupt these processes, leading to infertility or disorders such as PCOS, in which there is often an overproduction of pro-inflammatory cytokines^[33,34]^.

## Dysregulated immune responses in reproductive disorders

When the immune regulation mediated by monocytes becomes dysregulated, it can lead to various reproductive health issues. In conditions like endometriosis, monocytes secrete excessive amounts of pro-inflammatory cytokines, leading to chronic inflammation and the formation of ectopic endometrial tissue. This tissue can cause pain, adhesions, and infertility. Similarly, in PCOS, an overactive immune response can lead to insulin resistance, anovulation, and an increased risk of metabolic complications. In RPL, inadequate immune tolerance or excessive immune activation by monocytes can impair embryo implantation or lead to spontaneous abortion. Understanding how monocytes contribute to these conditions can help identify new therapeutic targets for improving reproductive health^[22,35]^.

## The role of monocyte-derived dendritic cells

Beyond their role as monocytes, the differentiation of monocytes into dendritic cells is also essential in reproductive health. Dendritic cells are key players in immune surveillance and the initiation of adaptive immune responses. In the reproductive tract, dendritic cells can recognize and present antigens to T-cells, influencing the immune tolerance necessary for embryo implantation. In pregnancy, dendritic cells derived from monocytes contribute to the maintenance of maternal–fetal tolerance by modulating T-cell responses and preventing immune rejection of the fetus. This underscores the importance of monocytes in regulating both the innate and adaptive immune systems in the context of reproduction^[36,37]^.

## Modulation of cytokine secretion as a therapeutic strategy

Given the critical role monocytes play in immune regulation, targeting their cytokine secretion presents a promising therapeutic strategy for managing reproductive health conditions. In disorders like endometriosis and PCOS, where cytokine dysregulation is prominent, therapies aimed at modulating monocyte-derived cytokines could offer relief. For example, anti-inflammatory treatments or cytokine inhibitors may help reduce chronic inflammation and improve fertility outcomes. In addition, therapies that enhance immune tolerance during pregnancy, such as immunomodulatory drugs, may help reduce the risk of pregnancy loss in women with autoimmune disorders or recurrent miscarriage^[38,39]^.

## Dysregulation of monocyte cytokine secretion in reproductive disorders

Dysregulation of monocyte cytokine secretion plays a crucial role in the pathogenesis of several reproductive disorders. Monocytes are key players in the immune system, known for their ability to secrete a wide range of cytokines that influence immune responses, inflammation, and tissue remodeling. During the menstrual cycle and pregnancy, the timely and balanced secretion of pro-inflammatory and anti-inflammatory cytokines by monocytes is critical for maintaining reproductive health. However, when this balance is disrupted, it can lead to the development or exacerbation of various reproductive disorders, including endometriosis, PCOS, RPL, and infertility^[13,40]^.

## Endometriosis

Endometriosis is a chronic, inflammatory condition characterized by the growth of endometrial-like tissue outside the uterus. Dysregulation of monocyte cytokine secretion is a key feature in the development and progression of this disease. Monocytes in women with endometriosis tend to produce higher levels of pro-inflammatory cytokines such as TNF-α, IL-1β, and IL-6. These cytokines not only promote chronic inflammation but also contribute to the growth and survival of ectopic endometrial tissue, leading to pain, adhesions, and infertility. Moreover, monocytes in endometriosis exhibit an altered phenotype, with an increased capacity to differentiate into macrophages that secrete additional pro-inflammatory mediators. The persistence of this inflammatory microenvironment is believed to impair immune tolerance, further exacerbating the disease and making it more resistant to treatment^[33,41–43]^.

## Polycystic ovary syndrome

In PCOS, an imbalance of monocyte cytokine secretion contributes to the pathophysiology of the disorder. Women with PCOS often exhibit an overproduction of pro-inflammatory cytokines, including IL-6, TNF-α, and IL-1β. These cytokines are implicated in the development of insulin resistance, a hallmark of PCOS, as well as ovarian dysfunction. The chronic low-grade inflammation observed in PCOS can lead to the disruption of normal follicular development, anovulation, and infertility. Additionally, the heightened inflammatory response can impair the response of the ovaries to gonadotropins, further compromising ovulation. The elevated cytokine levels may also contribute to the development of metabolic disturbances, such as obesity, that commonly coexist with PCOS^[44–46]^.

## Recurrent pregnancy loss

RPL is another reproductive disorder in which dysregulated monocyte cytokine secretion plays a significant role. In women with RPL, the maternal immune system fails to appropriately tolerate the presence of the embryo, leading to miscarriage. Monocytes in women with RPL often secrete higher levels of pro-inflammatory cytokines, such as TNF-α and IL-1β, which can negatively affect embryo implantation and early pregnancy development. This imbalance in cytokine secretion can disrupt the delicate immune tolerance required for successful pregnancy. Additionally, cytokines like IL-6 and IL-17 have been shown to impair trophoblast function, contributing to poor placental development and fetal loss. On the other hand, a deficiency in anti-inflammatory cytokines such as IL-10 and TGF-β can exacerbate the inflammatory response, further impairing maternal–fetal tolerance and increasing the risk of miscarriage. Modulating the cytokine profile of monocytes in women with RPL may provide a therapeutic approach to improving pregnancy outcomes^[16,47–49]^.

## Infertility

Infertility, particularly in women with unexplained causes, is often linked to an immune dysregulation in the reproductive tract. In these cases, monocytes may secrete abnormal amounts of cytokines that interfere with the reproductive process. Elevated levels of pro-inflammatory cytokines can disrupt endometrial receptivity and impair the ability of the uterus to support embryo implantation. In addition to affecting the endometrium, dysregulated cytokine secretion by monocytes can also interfere with ovarian function, disrupting the hormonal balance necessary for ovulation. Furthermore, an overactive immune response in the reproductive tract may lead to increased oxidative stress, which can damage both oocytes and sperm, contributing to infertility. Monocyte-mediated inflammation in the fallopian tubes and uterus can create an unfavorable environment for fertilization and embryo development, reducing the chances of successful conception^[18,50]^.

## Chronic inflammation and immune tolerance

The common thread among these reproductive disorders is chronic inflammation, which often results from the dysregulation of monocyte cytokine secretion. In conditions like endometriosis and PCOS, an overproduction of pro-inflammatory cytokines creates a persistent inflammatory state that disrupts normal reproductive function. In pregnancy, the inability of the maternal immune system to shift toward a tolerogenic response can lead to immune rejection of the embryo, resulting in miscarriage or pregnancy complications. Therefore, the ability to regulate monocyte cytokine secretion is crucial for maintaining immune tolerance and protecting reproductive health. Therapeutic strategies that target the inflammatory pathways mediated by monocytes and their cytokines hold promise for treating these conditions and improving fertility outcomes^[44,51]^.

## Therapeutic implications of cytokine modulation

The therapeutic modulation of cytokine secretion by monocytes offers significant potential in treating various reproductive disorders, where immune dysregulation plays a pivotal role. In conditions like endometriosis, PCOS, RPL, and infertility, cytokine imbalance – characterized by excessive pro-inflammatory cytokines or insufficient anti-inflammatory responses – disrupts normal reproductive function. Modulating these immune pathways could offer new therapeutic strategies for improving outcomes and enhancing fertility, by targeting the cytokines involved in chronic inflammation, immune tolerance, and tissue remodeling^[52,53]^.

## Targeting pro-inflammatory cytokines

One of the key strategies in treating immune-related reproductive disorders involves inhibiting the overproduction of pro-inflammatory cytokines, such as TNF-α, IL-1β, IL-6, and IL-17. These cytokines are frequently elevated in conditions like endometriosis and PCOS, where they contribute to chronic inflammation, ovarian dysfunction, and impaired endometrial receptivity. Monocyte-derived cytokines are critical drivers of the inflammatory response, and by reducing their secretion or activity, the inflammatory burden on reproductive tissues can be alleviated. Anti-cytokine therapies, such as monoclonal antibodies or small molecule inhibitors that block the action of TNF-α or IL-6, have already shown promise in treating inflammatory diseases such as rheumatoid arthritis and inflammatory bowel disease. These therapies may also be beneficial in treating endometriosis, where targeting TNF-α has been shown to reduce inflammation and improve fertility outcomes in some patients. For instance, TNF-α inhibitors, such as infliximab or adalimumab, are used in conditions involving chronic inflammation, and their application in reproductive disorders could help mitigate the inflammation associated with endometriosis and improve symptoms like pelvic pain and infertility. Similarly, IL-1β inhibitors such as anakinra have shown promise in reducing inflammation and improving reproductive outcomes in conditions with excessive inflammation. By targeting these cytokines, it is possible to restore immune balance and enhance fertility by reducing tissue damage, adhesion formation, and promoting endometrial receptivity^[54,55]^.

## Promoting anti-inflammatory cytokine secretion

While inhibiting pro-inflammatory cytokines is one approach, enhancing the production of anti-inflammatory cytokines, such as IL-10 and TGF-β, can also be a promising therapeutic strategy. These cytokines play critical roles in promoting immune tolerance, maintaining a favorable uterine environment for embryo implantation, and reducing tissue damage during the menstrual cycle. In conditions like RPL, where immune tolerance is impaired, boosting the production of these cytokines can help shift the immune system toward a more tolerogenic state, reducing the risk of miscarriage and improving pregnancy outcomes. Therapies that promote IL-10 or TGF-β production could involve the use of immune modulators or agents that activate the signaling pathways responsible for their synthesis. For example, IL-10 has been shown to inhibit the production of pro-inflammatory cytokines and promote tissue repair. Therapies aimed at enhancing the secretion of IL-10 could restore the delicate balance between immune activation and tolerance necessary for successful implantation and pregnancy. Additionally, promoting TGF-β signaling could help regulate immune responses and prevent excessive inflammation, improving fertility in patients with conditions like RPL and endometriosis^[56,57]^.

## Immunomodulatory drugs

Immunomodulatory drugs that regulate both pro-inflammatory and anti-inflammatory cytokine responses are another potential therapeutic avenue. These drugs aim to restore the immune system’s balance by either inhibiting the excessive inflammatory response or enhancing immune tolerance. For example, corticosteroids, which suppress a wide range of cytokine production, have been used in some cases of RPL to reduce inflammation and promote a more favorable immune environment for pregnancy. However, long-term use of corticosteroids is associated with potential side effects, such as adrenal suppression and increased infection risk, necessitating the development of more targeted therapies. Other immunomodulatory agents, such as methotrexate and hydroxychloroquine, have shown some promise in autoimmune-related reproductive disorders like endometriosis and RPL. These drugs may help regulate immune responses by inhibiting the activation of monocytes and reducing the secretion of pro-inflammatory cytokines. Moreover, newer classes of immunomodulatory drugs, such as Janus kinase inhibitors, which target specific signaling pathways involved in inflammation, may offer additional benefits in managing immune dysregulation in reproductive disorders^[58]^.

## Personalized medicine and cytokine modulation

A major challenge in cytokine modulation for reproductive health lies in the variability of immune responses among individuals. Not all women with conditions like endometriosis or RPL respond to the same treatment, and some may have a more robust or distinct immune profile than others. Personalized medicine, which tailors treatment based on the patient’s specific cytokine profile and immune responses, may provide more effective and safer therapeutic approaches. By identifying cytokine imbalances through biomarker profiling, clinicians could select the most appropriate treatments that target the specific cytokines or immune pathways involved in an individual’s reproductive disorder. Additionally, assessing the patient’s response to cytokine modulation therapies through monitoring cytokine levels and clinical outcomes (e.g., pain relief, improvement in fertility) would allow for the optimization of treatment regimens and minimize side effects. This personalized approach could lead to better-targeted therapies for managing conditions like endometriosis, PCOS, and RPL, improving the likelihood of success in restoring fertility and enhancing reproductive health^[59,60]^.

## Conclusion

Cytokine modulation, particularly through the regulation of monocyte cytokine secretion, holds great promise as a therapeutic strategy for improving reproductive health. Monocytes play a critical role in immune regulation, tissue remodeling, and inflammation during the menstrual cycle, and their cytokine secretions are central to both normal and pathological reproductive processes. Dysregulation of these cytokines can contribute to various reproductive disorders such as endometriosis, PCOS, RPL, and infertility. By targeting specific cytokines, either through inhibition of pro-inflammatory cytokines or promotion of anti-inflammatory cytokines, it may be possible to restore immune balance, reduce inflammation, and enhance fertility outcomes. While the potential for cytokine-based therapies is substantial, several challenges remain, including the need for personalized medicine approaches and a deeper understanding of the long-term effects of such therapies on reproductive health. Research into the complex interactions between immune cells, hormones, and cytokines during the menstrual cycle will be crucial for developing more targeted and effective treatments. Furthermore, therapeutic strategies must be carefully tailored to the individual, taking into account the unique immune profiles and reproductive conditions of each patient. With continued advancements in immunology and reproductive medicine, cytokine modulation offers a promising avenue for addressing immune-related reproductive disorders and improving fertility outcomes.

## Data Availability

Not applicable as this a perspective.

## Research registration unique identifying number (UIN)

Not applicable as this a perspective.
